# Remote Corticotomy Accelerates Orthodontic Tooth Movement in a Rat Model

**DOI:** 10.1155/2019/4934128

**Published:** 2019-06-17

**Authors:** Min Zou, Chenshuang Li, Zhong Zheng

**Affiliations:** ^1^Key Laboratory of Shaanxi Province for Craniofacial Precision Medicine Research, College of Stomatology, Xi'an Jiaotong University, Xi'an, Shaanxi 710004, China; ^2^Clinical Research Center of Shaanxi Province for Dental and Maxillofacial Diseases, College of Stomatology, Xi'an Jiaotong University, Xi'an, Shaanxi 710004, China; ^3^Department of Orthodontics, College of Stomatology, Xi'an Jiaotong University, Xi'an, Shaanxi 710004, China; ^4^Division of Growth and Development, Section of Orthodontics, School of Dentistry, University of California, Los Angeles, Los Angeles, CA 90095, USA

## Abstract

**Introduction:**

With an increasing demand for orthodontic treatment for adult patients, orthodontic professionals are constantly seeking novel strategies and technologies that can accelerate tooth movement in order to shorten the treatment period. For instance, in recent years, the influences of different surgical techniques on orthodontic tooth movement in the ipsilateral side of surgery were intensively investigated. Here, we attempt to examine if corticotomy could also affect the rate of tooth movement in the contralateral side of the surgery by using a rodent model.

**Materials and Methods:**

72 eight-week-old Sprague-Dawley rats were randomly divided into three groups as follows: the Control group (orthodontic treatment devices delivered only, no tooth movement), the orthodontic tooth movement (OTM) group (orthodontic treatment devices delivered and orthodontic treatment performed), and the Corticotomy + OTM group (remote corticotomy performed, orthodontic treatment devices delivered, followed by orthodontic treatment). The surgical procedure was conducted on the right side of the maxilla at the time of appliance placement and a force of 60 g was applied between the maxillary left first molar and maxillary incisors using nickel-titanium springs to stimulate OTM. The OTM distance and speed were tracked at 3, 7, 14, and 28 days post-surgery, followed by histological and immunohistochemical assessments.

**Results:**

In comparison with orthodontic treatment only, the contralateral corticotomy significantly accelerated OTM. Furthermore, animals undergoing corticotomy + OTM presented with a greater number of osteoclasts on the compression side, stronger staining of the osteogenic marker on the tension side, and higher expression of an inflammatory marker than the OTM group animals.

**Conclusion:**

Our current study demonstrates that remote corticotomy effectively accelerates alveolar bone remodeling and OTM. The study enriches our understanding of the regional acceleratory phenomenon (RAP) and offers an alternative strategy for accelerating OTM to shorten the orthodontic treatment period.

## 1. Introduction

Along with the functional and cosmetic improvements associated with orthodontic care, the duration of orthodontic treatment is a major factor that influences patient satisfaction. Due to the decreased metabolic activity in adults in comparison with children and adolescents, the prolonged orthodontic treatment becomes the top concern for adult patients. This problem is raising increasingly more attention since the number of adults seeking orthodontic treatment has increased considerably in the last 20 years [1]. Indeed, reducing the orthodontic treatment time is necessary for all patients to minimize the incidence of adverse effects such as root resorption, oral hygiene difficulties, and the appearance of caries [2, 3]. Therefore, promising strategies that could shorten orthodontic treatment are demanded by both patients and orthodontists [2, 4].

In response to this unmet need, low-intensity laser treatments, photobiomodulation, pulsed electromagnetic fields, corticotomy, corticision, and interseptal bone reduction have been investigated in attempts to reduce orthodontic treatment time [2]. Among these options, corticotomy is the most attractive approach since it is a common technique that induces regional acceleratory phenomenon (RAP) and expedites orthodontic tooth movement. However, it is not conclusive whether corticotomy is associated with accelerated orthodontic tooth movement clinically, as there are a plethora of controversial observations obtained from animal experiments and human clinical assessment [2, 4–6]. One plausible explanation for this contentious phenomenon is that although currently available investigations exclusively assessed tooth movement on the same side of surgery [2, 4–12], the controls used in animal and human studies were fundamentally different. In particular, in most animal studies, a separate group of animals without corticotomy was used as controls, while in human studies, tooth movement was compared between the side with corticotomy and the other side without surgery in the same patient [2, 4–6]. Considering that local and systemic immune responses may influence bone homeostasis during orthodontic treatment [13–17], it is possible that remote corticotomy can also accelerate tooth movement. If this is the case, the benefit of corticotomy on shortening orthodontic treatment may be underestimated in past human studies.

Here, we developed a novel rat model to test the aforementioned hypothesis and reconcile the discrepancies among previous studies. By performing a remote corticotomy before orthodontic treatment, we avoid the confounding effects of removing cortical bone blockage on the path of tooth movement which is a significant obstacle for previous corticotomy investigations [7–9, 12]. In addition, we have included a negative control group in the current study to eliminate the systematic error, in which only the orthodontic treatment devices were delivered, and no orthodontic force was applied to stimulate tooth movement. Thus, the current rat model is a proper model to evaluate the effects of remote corticotomy on orthodontic tooth movement.

## 2. Materials and Methods

### 2.1. Animal Model and Treatments

All animal surgeries were performed under the institutional protocol approved by the Animal Research Committee at Medical School of Xi'an Jiaotong University (No. XJTULAC2019-936) and are compliant with the Reporting In Vivo Experiments (ARRIVE) guidelines.

72 female Sprague-Dawley rats (8-week-old) were provided by the Experimental Animal Center, Medical School of Xi'an Jiaotong University, and were randomly divided into three treatment groups with four investigative time points (6 rats/treatment/time point) by a free online randomization tool at http://www.randomizer.org. The treatment groups are (1) the Control group - which wore orthodontic treatment devices only without tooth movement; (2) the orthodontic tooth movement (OTM) group - which wore orthodontic treatment devices and had orthodontic treatment; and (3) the Corticotomy + OTM group – which had undergone remote corticotomy, wore orthodontic treatment devices, and subsequently had orthodontic treatment. Rats were housed under controlled temperature (22 ± 1°C) in a 12-h/12-h light/dark cycle and were given free access to food and water.

**Figure 1:**
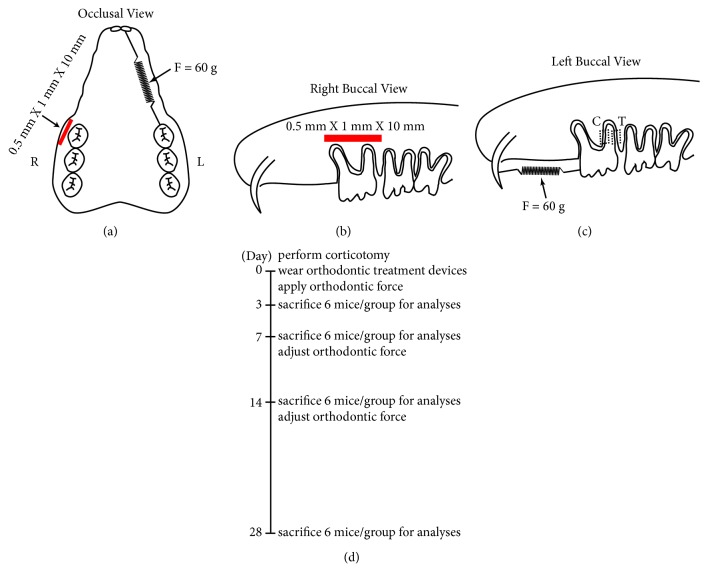
*Schematic drawing showing the experiment design for the Corticotomy + OTM group*. (a) Occlusal view, L: left, R: right. (b) Right buccal view. (c) Left buccal view, C: compression side, T: tension side. (d) The timeline of experimental performance.

A modified corticotomy procedure was performed in the current study. Briefly, after anesthesia by 10% chloral hydrate, a 15-mm horizontal full-thickness flap was made on the mucosa at the buccal vestibule at the right side of the maxilla. With the aid of a low-speed 1/4-mm spherical carbide bur, a size of 1-mm height, 10-mm length, and 0.5-mm depth cortical bone was removed at the level of root apex instead of several perforations [10–12] (Figures 1(a) and 1(b)). The surgery site was rinsed by sterile saline before suturing. No mobility of the hemimaxilla was detected after corticotomy. Tissues were subsequently sutured with absorbable thread. All surgical procedures were performed once only during the experimental period by the same well-trained D.D.S to ensure consistency.

Right after the surgery, the following procedures were performed on the rats from all three groups to wear the orthodontic equipment: a 0.5-mm wide horizontal cervical groove was made encircling the maxillary incisors, and a 0.020-inch stainless steel ligature wire was tightened around the groove. For the left maxillary first molar, another ligature wire was bent at the cervical margin of the tooth. These two wires on incisors and first molar were connected to a nickel-titanium coil spring (0.2 mm wire size, 1 mm in diameter and 1 mm in length; Smart Technology, Beijing, China) (Figures 1(a) and 1(c)).

For the OTM and Corticotomy + OTM groups, 60 g force was selected as it is a commonly used force level in comparable rat experiments [18–24]. The springs were strengthened to provide a force of 60 g [25] as confirmed by a force meter (YS-31 2N; YDM Corporation, Tokyo, Japan) to induce mesial movement of the upper left first molar (Figures 1(a) and 1(c)). The springs were fastened every week to ensure a consistent orthodontic force (Figure 1(d)).

All rats remained healthy during the experimental period. 6 rats from each group were sacrificed by 4% paraformaldehyde (PFA, Sigma-Aldrich, St Louis, Mo, USA) cardiac perfusion at day 3, 7, 14 or 28, respectively (Figure 1(d)). The maxilla from each rat was dissected, fixed with 4% ice-cold PFA for 24 hours, and decalcified by 19% ethylenediaminetetraacetic acid (Sigma-Aldrich) at room temperature for 2 months before paraffin embedding.

### 2.2. Measurement of Orthodontic Tooth Movement

For each individual rat, silicon rubber impressions of maxillary dentition were taken and cast into an ultra-anhydrite plaster model after euthanasia. The models were labeled by random numbers and sent to three independent observers for measurement in a blinded fashion. The distance between mesial pits of upper first molars and upper third molars on both sides for each sample was measured three times by an individual observer using digital calipers. The orthodontic tooth movement distance for each sample is defined as the following formula:

(1) $$\begin{array}{c} D_{OTM}=L_{m1-m3}-R_{m1-m3}^{\prime } \end{array}$$

D_OTM_: the orthodontic tooth movement distance

L_m1−m3_: the distance between mesial pits of upper left first molar and upper left third molar;

R_m1−m3_′: the mean of the results of three measurements of R_m1−m3_;

R_m1−m3_: the distance between mesial pits of upper right first molar and upper right third molar.

At both the left and right sides of each sample, the moving distances were measured by an individual observer three times. The average of the mean values obtained by three independent observers was calculated for further data analyses.

### 2.3. Histological and Immunohistochemical Analyses

Serial axial 5-*μ*m sections were obtained from each paraffin embedded samples for hematoxylin and eosin (H&E), tartrate-resistant acid phosphatase (TRAP), and immunofluorescent staining as previously described [13, 16]. Images were acquired at room temperature under a microscope (Olympus, America Inc., Center Valley, PA, USA) using a 20× (dry HC Plan Apochromat, NA 0.17) objective lens.

Particularly, TRAP staining was performed per the manufacturer's instructions (386-A1, Sigma-Aldrich) to identify osteoclasts [12, 26]. The numbers of osteoclasts per observation field (each image under 20X objective lens) were determined on the alveolar bone surface and marrow spaces adjacent to the mesial surfaces (compression side) of distobuccal roots according to methods previously described [12, 26]. Marrow spaces were selected only if they were adjacent to the alveolar bone surface. Four sections that revealed the most pulp structure (mid-root sections) were used for measurements, and their means were used for statistical tests.

The expression levels of osteocalcin (OCN), a marker of alveolar bone anabolic activity [27, 28], and interleukin 6 (IL6), a known essential immune marker during orthodontic tooth movement [14, 15, 27, 28] at different time points, were evaluated by immunofluorescent staining. Anti-osteocalcin (OCN) and anti-IL6 primary antibodies were purchased from Abcam (Cambridge, MA, USA). 4′,6-Diamidino-2-phenylindole (DAPI; Sigma-Aldrich) was used for nuclear counterstaining.

### 2.4. Statistical Analysis

Statistical analysis was performed with OriginPro® 8 (Origin Lab Corp., Northampton, MA, USA) that included Mann-Whitney U test, one-way ANOVA test, and two-sample* t*-test.* P *< 0.05 was considered a significant difference.

## 3. Results

### 3.1. Corticotomy Increased the Moving Distance and Speed of Orthodontic Teeth on the Contralateral Side

**Figure 2:**
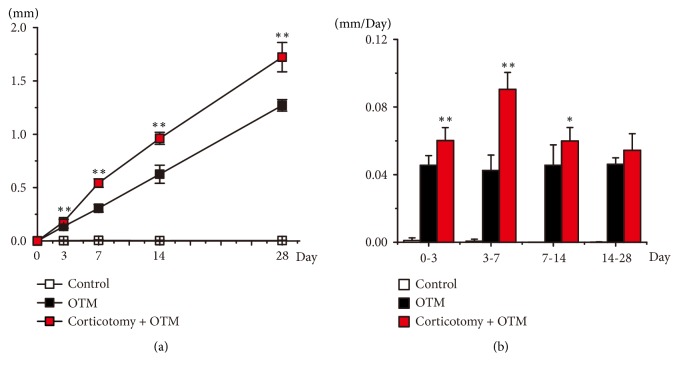
*Corticotomy increased the moving rate and distance of the orthodontic teeth on the contralateral side*. (a) The distance and (b) speed of orthodontic teeth movement during the 28-day experimental period. N = 6. Two sample* t*-test was used for statistical analysis. *∗*:* P *< 0.05 when compared with the OTM group; *∗∗*:* P *< 0.005 when compared with the OTM group.

Since there was no orthodontic force applied, orthodontic teeth in Control group should not move at all. As expected, the upper left first molars in the Control group did not move during the entire experimental period (Figure 2(a)), representing the accuracy of the measurement system. Application of orthodontic force led to the mesial moving of the upper left first molars gradually as seen in the OTM group as well as the Corticotomy + OTM group (Figure 2(a)). More importantly, the moving distance of the orthodontic teeth in the Corticotomy + OTM group was significantly longer than that in the OTM group (Figure 2(a)). Particularly, after seven days of applying orthodontic force, the moving distance in the Corticotomy + OTM group was almost 1.5-fold as large as that in the OTM only group (Figure 2(a)).

In order to track the tooth movement in detail, the average moving speed of the orthodontic teeth between every two investigating time points was calculated (Figure 2(b)). Again, the movement of orthodontic teeth in the Control group was found to be negligible (at a speed slower than 0.005 mm/Day). Unlike orthodontic teeth in the OTM group that moved at a consistent speed (approximately 0.045 mm/day), the orthodontic teeth in the Corticotomy + OTM group had significantly accelerated movement during day 0 to 7, during which its moving speed was 1.5-fold of that in the OTM group among day 0-3 (approximately 0.060 mm/day) and 2-fold of that in the OTM group during day 3-7 (approximately 0.090 mm/day). In day 7-14, the moving speed of orthodontic teeth in Corticotomy + OTM group was considerably reduced from the peak seen in day 3-7 but was still significantly higher (approximately 0.060 mm/day) than that of the OTM group. The rate of movement of teeth in the Corticotomy + OTM group subsequently slowed down to a similar level to that of the OTM group during day 7-14 (approximately 0.055 mm/day) (Figure 2(b)).

### 3.2. Remote Corticotomy Accelerated Alveolar Bone Remodeling around the Orthodontic Teeth

**Figure 3:**
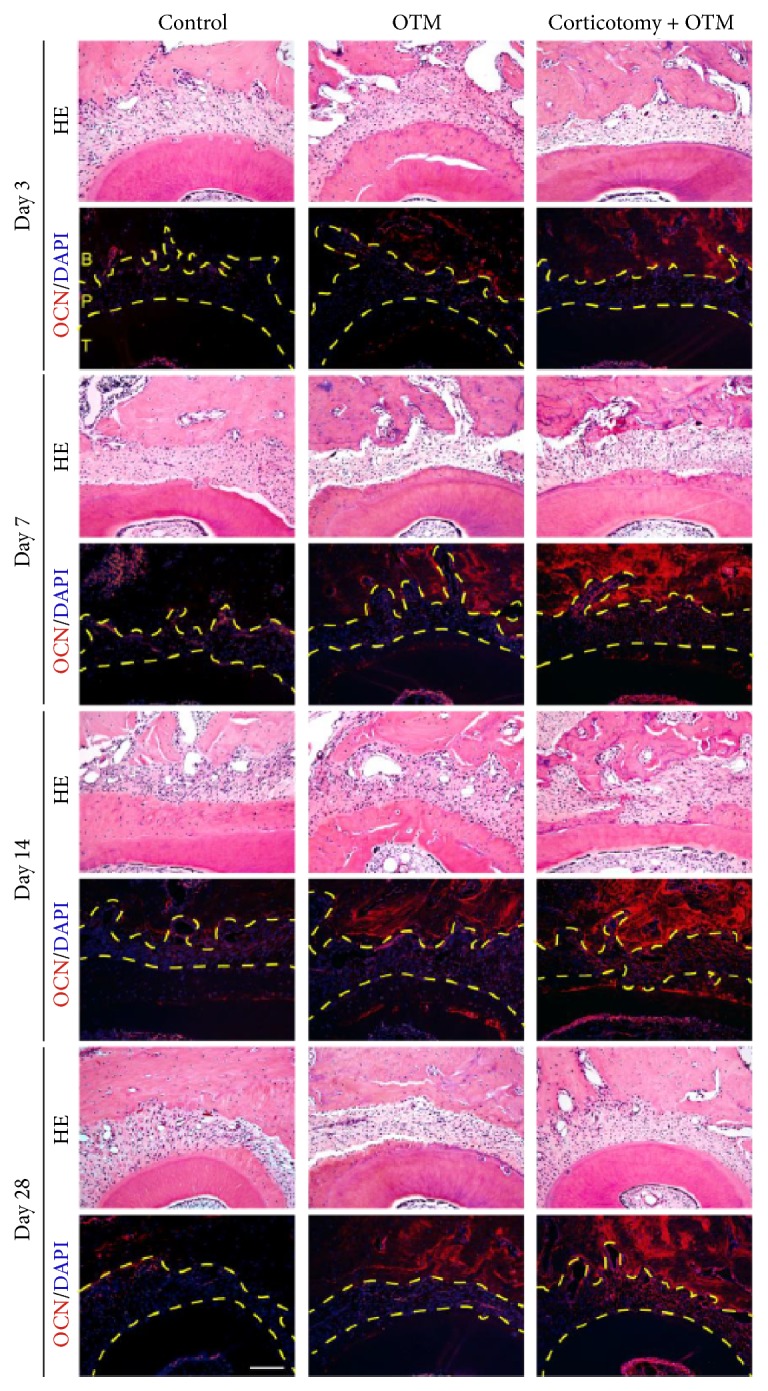
*Corticotomy increased Osteocalcin expression in the periodontal tissue on the tension side of the orthodontic teeth*. The immunofluorescent staining of osteocalcin (OCN) in the periodontal tissue around the distal surface of the distal buccal root of orthodontic teeth on day 3, day 7, day 14, and day 28. Larger staining positive area was noticed in the Corticotomy + OTM group at day 7, day 14, and day 28 when compared to that in the OTM group. B: alveolar bone; P: periodontal ligament; T: tooth. Scale bar = 100 *μ*m.

To assess the bone anabolic activity in the tension side of the orthodontic teeth, the expression levels of OCN were detected by immunofluorescent staining (Figure 3). In the Control group, there was no obvious positive signal of OCN along the edge of alveolar bone on the tension side of the distobuccal roots of the upper left first molars as expected (Figure 3). While only weak and discontinuous staining of OCN was found in the respective region of the OTM group (Figure 3), OCN staining was apparently detected in the respective region of the Corticotomy + OTM group (Figure 3). The levels of OCN in the Corticotomy + OTM group were significantly higher than those in the OTM group on days 7, 14, and 28 (Figure 3).

**Figure 4:**
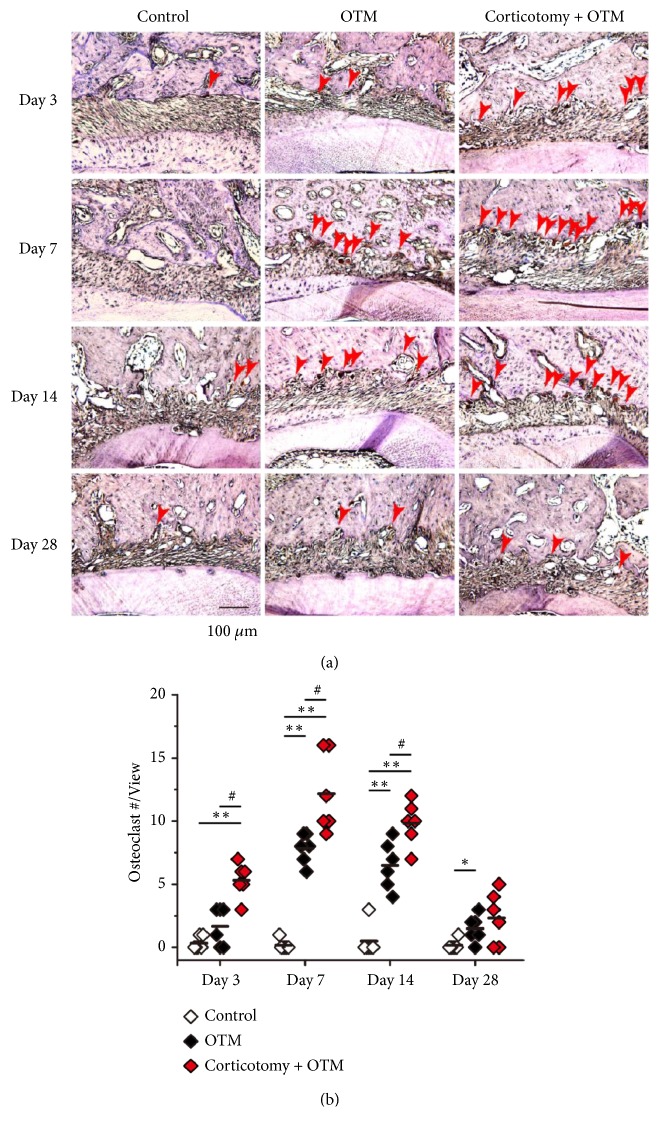
*Corticotomy increased the osteoclast number in the periodontal tissue on the compression side of the orthodontic teeth*. (a) The TRAP staining in the periodontal tissue around the mesial surface of the distal buccal root of orthodontic teeth. Red solid arrows: osteoclasts with TRAP staining. (b) The number of osteoclasts in the periodontal tissue around the mesial surface of the distal buccal root of orthodontic teeth per observation field. N = 6. Mann-Whitney test was used for statistical analysis. *∗*:* P *< 0.05 when compared with the Control group. *∗∗*:* P *< 0.005 when compared with the Control group. #:* P *< 0.05 when compared with the OTM group.

In the compression side, the bone metabolic activity was evaluated by the number of osteoclasts which were detected by TRAP staining. In the Control group, the surface of the alveolar bone remained smooth and there was no obvious osteoclast recruitment (Figure 4(a)). On day 3, an average of 1.7 osteoclasts per observation field was detected at the surface of the alveolar bone on the compression side of the orthodontic teeth in the OTM group, while the number of osteoclasts increased to 7.8 per observation field on day 7 and then decreased to 6.5 at day 14 and 1.5 on day 28 (Figures 4(a) and 4(b)). From day 3 to day 14, the number of osteoclasts in the Corticotomy + OTM group was kept at a significantly higher level when compared to the OTM group, while the density of osteoclasts was similar in both OTM and Corticotomy + OTM group on day 28 (Figures 4(a) and 4(b)).

### 3.3. Remote Corticotomy Enhanced the Immune Response in the Periodontal Tissue around the Orthodontic Teeth

**Figure 5:**
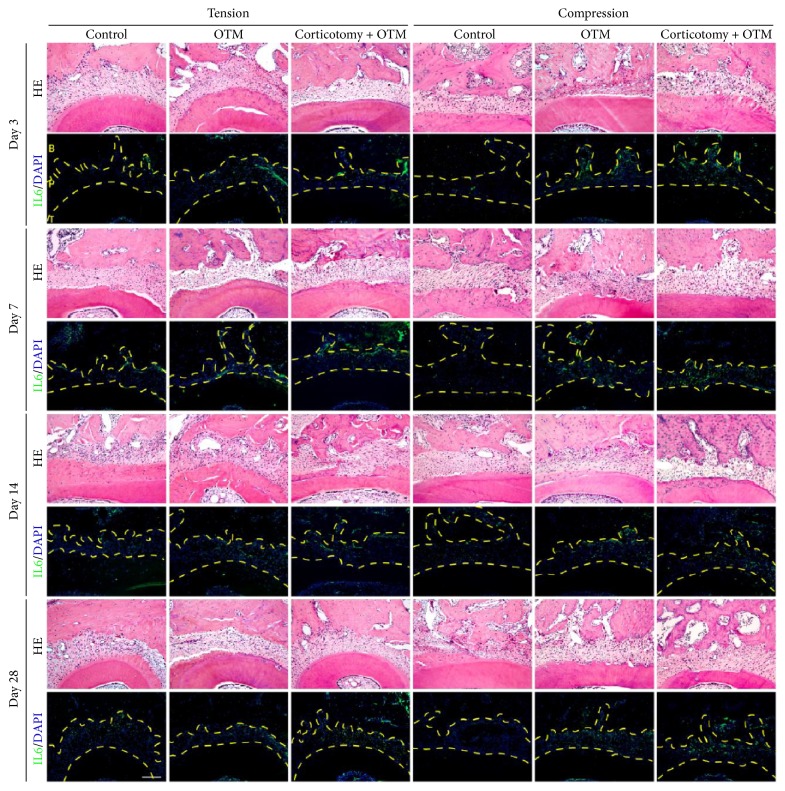
*Corticotomy increased IL6 expression in the periodontal tissue around the orthodontic teeth*. The immunofluorescent staining of IL6 in the periodontal tissue around the distal buccal root of orthodontic teeth on day 3, day 7, day 14, and day 28. OTM group has stronger staining intensity when compared to the Control group. The Corticotomy + OTM group has the strongest staining intensity at day 7 when compared to other groups. B: alveolar bone; P: periodontal ligament; T: tooth. Scale bar = 100 *μ*m.

In order to explore the underlying mechanism, we tested the expression of IL6, a known essential immune marker during orthodontic tooth movement [14, 15, 29, 30]. In the Control group, no obvious IL6 staining was observed in the periodontal tissue around the orthodontic teeth - upper left first molars, while IL6 staining was found in the appropriate area in both the OTM and the Corticotomy + OTM groups (Figure 5). Interestingly, the higher intensity of IL6 staining could be observed on both the compression and the tension sides in the Corticotomy + OTM group compared to the OTM group (Figure 5).

## 4. Discussion

The stimulus and RAP are known to be proportional; thus, the larger the noxious stimulus, the greater the RAP [31]. For instance, when bone is injured, the adjacent hard and soft tissues would actively reconstruct [31]. Nevertheless, RAP was found to be a complex physiological process that could accelerate bone healing [32–34]. Recent studies have indicated that RAP also contributes to accelerating orthodontic tooth movement after orthognathic surgery [35], although the further in-depth mechanical explanation is pending.

In contrast to previously established models, in which corticotomy was performed around the orthodontic tooth and/or on the moving path of the orthodontic tooth [8, 12, 36], here, corticotomy was performed on the contralateral side of the treated teeth to avoid the confounding effects of removing cortical bone blockage during orthodontic tooth movement. By using this model, we observed increased osteoclast recruitment on the compression side accompanied by elevated OCN expression on the tension side of the orthodontic teeth in the Corticotomy + OTM group when compared to those in the OTM group. These phenomena indicated that, despite the similar pattern of periodontal tissue remodeling, the remote corticotomy significantly enhanced the rate of the remodeling process and resulted in the accelerated orthodontic tooth movement. Therefore, the influence of remote corticotomy should be considered when interpreting human studies that compare orthodontic tooth movement in the surgical side to that of the contralateral side without surgery of the same patient.

Mechanically, not only the local aseptic inflammation is associated with alveolar bone remodeling [37–39], the systemic immune responding to bone homeostasis is also involved [40]. Especially, previous studies also suggested that immune cells, such as M1-like macrophages and T cells, and inflammatory cytokines play important roles in promoting orthodontic tooth movement [13, 16, 37–39]. Aligning with previous studies that show that OTM induces the systemic immune response [17], we found higher expression levels of IL6 around the orthodontic teeth when orthodontic treatment was performed in comparison with the Control group. And* vice versa*, we also noticed that remote corticotomy further enhanced IL6 expression in the periodontal tissue around orthodontic teeth on the contralateral side of corticotomy, which indicates that the immune response in the Corticotomy + OTM group is stronger than that in the OTM only group. As the surgery site was far away from the orthodontic tooth in the current animal model, this IL6 increase is not likely due to the effects of the local immune response. Therefore, our data strongly support the hypothesis that the activation of the systemic immune response could accelerate alveolar bone remodeling and orthodontic tooth movement. Moreover, identifying the benefits of remote corticotomy on orthodontic tooth movement may help reconcile the e conflicting data of the efficacy of corticotomy, while further investigation is warranted to unveil the underlying mechanics. As rats have shorter lifetimes when compared to humans, further verification in pre-clinical large animal models and clinical investigations with a large patient population is needed to verify our current findings.

## 5. Conclusion

In this study, we have developed a new rat model to investigate the effects of corticotomy in orthodontic treatment. We confirm that corticotomy can accelerate orthodontic tooth movement and alveolar bone remodeling on the contralateral side of the surgical procedure. This investigation potentially enriches our choices for shortening the duration of orthodontic treatment in the clinical setting.

## Data Availability

The data used to support the findings of this study are included in the article.

## References

[1] Jawad Z., Bates C., Hodge T. (2015). Who needs orthodontic treatment? Who gets it? and who wants it?. *British Dental Journal*.

[2] Gkantidis N., Mistakidis I., Kouskoura T., Pandis N. (2014). Effectiveness of non-conventional methods for accelerated orthodontic tooth movement: a systematic review and meta-analysis. *Journal of Dentistry*.

[3] Shoreibah E. A., Ibrahim S. A., Attia M. S., Diab M. M. (2012). Clinical and radiographic evaluation of bone grafting in corticotomy-facilitated orthodontics in adults. *Journal of the International Academy of Periodontology*.

[4] Fernandez-Ferrer L., Montiel-Company J., Candel-Marti E., Almerich-Silla J., Penarrocha-Diago M., Bellot-Arcis C. (2016). Corticotomies as a surgical procedure to accelerate tooth movement during orthodontic treatment: A systematic review. *Medicina Oral Patología Oral y Cirugia Bucal*.

[5] Kalemaj Z., Debernardl C. L., Buti J. (2015). Efficacy of surgical and non-surgical interventions on accelerating orthodontic tooth movement: A systematic review. *European Journal of Oral Implantology*.

[6] Long H., Pyakurel U., Wang Y., Liao L., Zhou Y., Lai W. (2013). Interventions for accelerating orthodontic tooth movement: a systematic review. *The Angle Orthodontist*.

[7] Abbas N. H., Sabet N. E., Hassan I. T. (2016). Evaluation of corticotomy-facilitated orthodontics and piezocision in rapid canine retraction. *American Journal of Orthodontics and Dentofacial Orthopedics*.

[8] Iglesias-Linares A., Yañez-Vico R. M., Moreno-Fernandez A. M., Mendoza-Mendoza A., Solano-Reina E. (2012). Corticotomy-assisted orthodontic enhancement by bone morphogenetic protein-2 administration. *Journal of Oral and Maxillofacial Surgery*.

[9] Kurohama T., Hotokezaka H., Hashimoto M. (2017). Increasing the amount of corticotomy does not affect orthodontic tooth movement or root resorption, but accelerates alveolar bone resorption in rats. *European Journal of Orthodontics*.

[10] Librizzi Z., Kalajzic Z., Camacho D., Yadav S., Nanda R., Uribe F. (2017). Comparison of the effects of three surgical techniques on the rate oforthodontic tooth movement in a rat model. *The Angle Orthodontist*.

[11] Peron A. P., Johann A. C., Papalexiou V. (2017). Tissue responses resulting from tooth movement surgically assisted by corticotomy and corticision in rats. *The Angle Orthodontist*.

[12] Wang L., Lee W., Lei D., Liu Y., Yamashita D., Yen S. L. (2009). Tisssue responses in corticotomy- and osteotomy-assisted tooth movements in rats: histology and immunostaining. *American Journal of Orthodontics and Dentofacial Orthopedics*.

[13] He D., Kou X., Yang R. (2015). M1-like macrophage polarization promotes orthodontic tooth movement. *Journal of Dental Research*.

[14] León-Romero L. C., Rodríguez-Orozco A. R., De La Luz Vargas-Purecko M., Ruiz-Reyes H. (2012). Saliva components reestablish the basal production of IL-6 by mononuclear cells, 72 hours after nitinol archiwire placement: A preliminary study. *Iranian Journal of Allergy, Asthma and Immunology*.

[15] Madureira D. F., Taddei S. D. A., Abreu M. H. N. G., Pretti H., Lages E. M. B., Da Silva T. A. (2012). Kinetics of interleukin-6 and chemokine ligands 2 and 3 expression of periodontal tissues during orthodontic tooth movement. *American Journal of Orthodontics and Dentofacial Orthopedics*.

[16] Yan Y., Liu F., Kou X. (2015). T cells are required for Orthodontic tooth movement. *Journal of Dental Research*.

[17] Zeng M., Kou X., Yang R. (2015). Orthodontic force induces systemic inflammatory monocyte responses. *Journal of Dental Research*.

[18] Wei X. X., Chu J. P., Zou Y. Z., Ru N., Cui S. X., Bai Y. X. (2015). Effect of odanacatib on root resorption and alveolar bone metabolism during orthodontic tooth movement. *Genetics and Molecular Research*.

[19] Shirazi M., Ahmad Akhoundi M. S., Javadi E. (2013). The effects of diode laser (660 nm) on the rate of tooth movements: an animal study. *Lasers in Medical Science*.

[20] Shirazi M., Ameri A., Shafaroodi H. (2008). Orthodontic tooth movement in cholestattic and cirrhotic rats. *Journal of Orthodontics*.

[21] Nilforoushan D., Shirazi M., Dehpour A.-R. (2002). The role of opioid systems on orthodontic tooth movement in cholestatic rats. *The Angle Orthodontist*.

[22] Shirazi M., Nilforoushan D., Alghasi H., Dehpour A.-R. (2002). The role of nitric oxide in orthodontic tooth movement in rats. *The Angle Orthodontist*.

[23] Shirazi M., Khosrowshahi M., Dehpour A.-R. (2001). The effect of chronic renal insufficiency on orthodontic tooth movement in rats. *The Angle Orthodontist*.

[24] Bridges T., King G., Mohammed A. (1988). The effect of age on tooth movement and mineraldensity in the alveolar tissues of the rat. *American Journal of Orthodontics and Dentofacial Orthopedics*.

[25] King G. J., Keeling S. D., McCoy E. A., Ward T. H. (1991). Measuring dental drift and orthodontic tooth movement in response to various initial forces in adult rats. *American Journal of Orthodontics and Dentofacial Orthopedics*.

[26] Ren Y., Kuijpers-Jagtman A. M., Maltha J. C. (2005). Immunohistochemical evaluation of osteoclast recruitment during experimental tooth movement in young and adult rats. *Archives of Oral Biolog*.

[27] Baloul S. S., Gerstenfeld L. C., Morgan E. F., Carvalho R. S., van Dyke T. E., Kantarci A. (2011). Mechanism of action and morphologic changes in the alveolar bone in response to selective alveolar decortication-facilitated tooth movement. *American Journal of Orthodontics and Dentofacial Orthopedics*.

[28] Franzen T. J., Monjo M., Rubert M., Vandevska-Radunovic V. (2014). Expression of bone markers and micro-CT analysis of alveolar bone during orthodontic relapse. *Orthodontics & Craniofacial Research*.

[29] d'Apuzzo F., Cappabianca S., Ciavarella D., Monsurrò A., Silvestrini-Biavati A., Perillo L. (2013). Biomarkers of periodontal tissue remodeling during orthodontic tooth movement in mice and men: overview and clinical relevance. *The Scientific World Journal*.

[30] Uematsu S., Mogi M., Deguchi T. (1996). Interleukin (IL)-1*β*, IL-6, tumor necrosis factor-*α*, epidermal growth factor, and *β*2-microglobulin levels are elevated in gingival crevicular fluid during human orthodontic tooth movement. *Journal of Dental Research*.

[31] Frost H. M. (1983). The regional acceleratory phenomenon: a review. *Henry Ford Hospital Medical Journal*.

[32] Frost H. M. (1989). The biology of fracture healing. An overview for clinicians. Part I. *Clinical Orthopaedics and Related Research*.

[33] Frost H. M. (1989). The biology of fracture healing. An overview for clinicians. Part II. *Clinical Orthopaedics and Related Research*.

[34] Yaffe A., Fine N., Binderman I. (1994). Regional accelerated phenomenon in the mandible following mucoperiosteal flap surgery. *Journal of Periodontology*.

[35] Liou E. J. W., Chen P.-H., Wang Y.-C., Yu C.-C., Huang C. S., Chen Y.-R. (2011). Surgery-first accelerated orthognathic surgery: Postoperative rapid orthodontic tooth movement. *Journal of Oral and Maxillofacial Surgery*.

[36] Yuan H., Zhu X., Lu J., Dai J., Fang B., Shen S. G. F. (2014). Accelerated orthodontic tooth movement following le Fort i osteotomy in a rodent model. *Journal of Oral and Maxillofacial Surgery*.

[37] Alhashimi N., Frithiof L., Brudvik P., Bakhiet M. (2000). Orthodontic movement induces high numbers of cells expressing IFN-*γ* at mRNA and protein levels. *Journal of Interferon & Cytokine Research*.

[38] Garlet T. P., Coelho U., Silva J. S., Garlet G. P. (2007). Cytokine expression pattern in compression and tension sides of the periodontal ligament during orthodontic tooth movement in humans. *European Journal of Oral Sciences*.

[39] Ren Y., Hazemeijer H., de Haan B., Qu N., de Vos P. (2007). Cytokine profiles in crevicular fluid during orthodontic tooth movement of short and long durations. *Journal of Periodontology*.

[40] Arron J. R., Choi Y. (2000). Bone versus immune system. *Nature*.

